# Application progress of magnetic resonance imaging in thyroid associated ophthalmopathy

**DOI:** 10.3389/fendo.2025.1537957

**Published:** 2025-06-26

**Authors:** Yan Song, Tuo Li, Wei Tang, Qian Lv, Xing-xing Zhang, Wei-yi Zhou, Yong-quan Shi

**Affiliations:** Department of Endocrinology and Metabolism, Changzheng Hospital, Naval Medical University, Shanghai, China

**Keywords:** thyroid associated ophthalmopathy, magnetic resonance imaging, signal intensity ratio, T2 mapping, diffusion-weighted imaging

## Abstract

Thyroid associated ophthalmopathy (TAO) is an autoimmune orbital disease associated with thyroid dysfunction, which may significantly impact the appearance and quality of life of patients. The course of TAO includes active stage and inactive stage, and the treatment methods are different in different stages. The clinical activity score is often used to evaluate the activity of TAO, but it is easy to be affected by subjective factors because it is based on the symptoms and signs of patients. Magnetic resonance imaging (MRI) can evaluate the postorbital tissue structure of TAO more objectively and accurately. A variety of MRI modalities have been used in the diagnosis and treatment of TAO. This article mainly summarizes the application of MRI in diagnosing TAO, assessing its activity, predicting treatment efficacy, and evaluating therapeutic outcomes, thereby providing clinicians with additional evidence-based options.

## 1 Introduction

Thyroid associated ophthalmopathy (TAO) is an organ-specific autoimmune disorder resulting from multifactorial etiologies, characterized by extensive ocular inflammation and dysfunction of the extraocular muscles (EOMs) (1). Enlargement of EOMs constitutes the most significant pathological alteration observed in the orbits of patients with TAO (2). Current evidence suggests that orbital fibroblasts are the target cells of TAO, which secrete large amounts of hyaluronic acid under the action of various cytokines (3), and can also differentiate into mature adipocytes (4). These cellular changes lead to the characteristic enlargement of EOMs and postorbital fat dilation in TAO. In the chronic stage of the disease, collagen deposition, fibroblast proliferation, and fibroplasia occur within the affected EOMs. The clinical manifestations of TAO include mild ocular discomfort, eyelid retraction, exophthalmos, diplopia, and compressive optic neuropathy. It not only affects the patient’s appearance and quality of life, but may also result in blindness in severe cases (5). Therefore, early diagnosis and treatment are crucial for alleviating patients’ symptoms.

In the clinical course of TAO, two distinct stages of disease activity have been observed: the early active stage and the late inactive stage (6). In the active stage, the posterior orbital tissue is edematous, and histological examination showed a large number of lymphocyte and plasma cell infiltration. In the inactive stage, the posterior orbital tissue is characterized by fibrosis (7). The length of the active period varies from several months to several years. Immunomodulatory therapy mainly targets edema, lymphocyte infiltration, and activated fibroblasts. Thus, immunotherapy is more effective during the active stage, while the therapeutic effect will decrease during the inactive stage. The evaluation of TAO activity is absolutely necessary for the selection of disease treatment and the prediction of medical management effects. TAO can be assessed according to the clinical activity score(CAS) and the severity grading identified by the European Group on Graves’ Orbitopathy (EUGOGO) (8). However, the scoring and grading criteria are based on the symptoms and signs of patients, which are easily affected by the clinical experience of physicians and the state of patients, so more objective and reliable parameters are needed to evaluate the condition.

Magnetic resonance imaging(MRI) is the most commonly used imaging method in TAO. MRI has important advantages such as nonionizing radiation, high inherent soft tissue contrast, better reflection of tissue water content, and the ability to quantify multiple parameters (9). TAO mainly involves the changes of soft tissues such as EOMs and adipose tissue in orbit. MRI can provide objective measurement parameters to help physicians better assess the TAO condition, especially for some early stage patients with mild symptoms. In recent years, MRI has achieved substantial advancements in the diagnosis, evaluation, and prediction of therapeutic outcomes for TAO. Numerous studies have elucidated pathological changes, such as inflammation and fibrosis of retroorbital tissues, via quantitative analysis of orbital MRI, thereby enabling earlier and more precise detection of microstructural alterations in ocular soft tissues (9–11). This article provides a comprehensive review of the research progress and prospective application of MRI in the diagnosis and management of TAO. Currently, research primarily focuses on TAO diagnosis, differentiation between active and inactive phases, therapeutic effect prediction, and post-treatment efficacy assessment. We systematically outline the applicable MRI sequences and measurement parameters for each phase to assist clinicians in optimizing the diagnosis and treatment of TAO.

## 2 Application of MRI in diagnosis and activity evaluation of TAO

### 2.1 Application of MRI in the diagnosis

According to the Bartley diagnostic criteria of TAO (12), exophthalmos, EOMs and optic nerve involvement are all important evaluation indicators. MRI is one of the most commonly used imaging in clinical evaluation of EOMs, optic nerve and exophthalmos. Especially for TAO patients with only minor eye discomfort and no obvious signs, MRI examination is more necessary to confirm the diagnosis. The measurement of retroorbital tissue parameters using MRI sequences is helpful for the diagnosis of TAO (**Table 1**).

**Table 1:** MRI sequences applied in TAO diagnosis.

Tissue	Clinical features	MRI parameter/sequence	Advantages
EOMs	Enlargement	diameter, cross-sectional area and volume	Objectively evaluate the changes of the extraocular muscles and discover the early microstructure changes
Eyeball	Exophthalmos	the perpendicular distance between the interzygomatic line and the surface of the cornea	Objectively measure the degree of proptosis to reduce measurement errors
Optic nerve	DON	diameter,area, volume, T2RT,WF, ReHo, IDEAL-T2WI,DTI, T2 Mapping, etc	Predict and detect early optic nerve lesions and assist in diagnosis

EOMs, extraocular muscle; DON, dysthyroid optic neuropathy; T2RT, T2 relaxation time; WF, water fractio; ReHo, regional homogeneity; IDEAL-T2WI, iterative decomposition of water and fat with echo asymmetry and least-squares estimation-T2-weighted images; DTI, diffusion tensor imaging.

Enlargement and volume changes of EOMs are common in TAO. MRI can clearly show the morphology of the EOMs and further measure the diameter, cross-sectional area and volume. A basic rule for MRI is that T1- weighted(T1w) images are best for anatomic structure, while T2-weighted (T2w) sequences give more information about the different tissue composition. The advantage of MRI is the ability to view several (axial, coronal, sagital) angles without movement of the patient. T1w axial and coronal sequences give an overview of the extent of the disease, concerning globe proptosis and involvement of the different recti muscles. The thickness of the medial and lateral rectus muscles should be measured on axial images, and the thickness of the superior and inferior rectus muscles should be measured on coronal images. Major morphological diagnostic criteria include a spindle-like spreading of the recti muscles (>4 mm) without involvement of the tendon, a compression of the optic nerve in the orbital apex (crowded orbital apex syndrome) and the absence of any space occupying intraorbital process (7, 13). One study showed that the thickness of EOMs had a significant correlation with cross-sectional area, but a weak correlation with muscle volume, suggesting that the measurement of EOMs volume cannot be simply replaced by thickness (14). The conventional MRI method for measuring the volume of the EOMs is to obtain the volume of each layer by multiplying the layer thickness by the cross-sectional area of each layer, and then adding all the calculated volumes to obtain the total volume of the EOMs (15, 16). Now, the volume of the EOMs can also be measured by three-dimensional(3D) reconstruction. Shen et al. used Mimics software for 3D reconstruction based on orbital MRI data, and confirmed that the orbital soft tissue volume calculated based on this method is both reliable and accurate (17). Tang et al. investigated the accuracy of two semi-automatic segmentation measurements based on MRI 3D Cube fast spin echo (FSE)-flex sequence in phantoms, and evaluated the feasibility of semi-automatic segmentation for the determination of the volumetric alterations of orbital fat and total EOMs in patients with TAO. They found that MRI Cube FSE-flex determined multi-dimensional threshold is a relatively accurate semi-automatic segmentation that can be used to evaluate orbital fat and total EOMs volumes in clinic (18). Therefore, parameters such as the diameter, cross-sectional area and volume of the EOMs measured by MRI can be used to assist in the diagnosis of TAO.

Exophthalmos, a common clinical manifestation of TAO, is defined as being 3mm larger than the upper limit of the normal range, which is helpful in assessing the severity of TAO and response to treatment (19). Traditionally, the Hertel exophthalmometer is commonly used to measure the ocular protrusion, but it is easily affected by the experience and proficiency of testing physicians, so the accuracy of the data is poor. MRI can objectively display images of exophthalmos, and the measurement data is more reliable. For this, the axial slice that most obviously depicts the EOMs and optic nerve is selected, and from which the perpendicular distance between the interzygomatic line and the surface of the cornea is measured (13, 20).

Dysthyroid optic neuropathy (DON) is the most serious complication in TAO, with an incidence of 3-5% (21). The mechanism of DON is based on orbital apex crowding due to the enlargement of soft tissues, including the EOMs and fat (22, 23). The existing diagnostic criteria for DON are primarily based on clinical signs and symptoms, which are neither sensitive nor specific. Barrett et al. suggested that imaging is particularly important for the evaluation of DON (24). Previous imaging studies mainly used apical crowding signs (enlargement of EOMs, fat volume, and orbital bony geometry) to detect DON. However, EUGOGO’s research demonstrated that DON may also occur in TAO patients without enlarged extraocular muscles. Therefore, studying the optic nerve is more valuable for DON diagnosis. EUGOGO recommended that clinical features, imaging examinations, and electrophysiological visual examination, can be used for the diagnosis of DON (23). Recently, more MRI indicators have been used to detect DON, including the presence of fluid in the optic nerve sheath, muscle index, optic nerve diameter, optic nerve stretching or apical crowding, etc (25–28). Dodds et al. used high-resolution MRI to measure the diameter of the optic nerve in multiple planes. They found that the radial diameters of the optic nerve are useful for predicting DON risk (29). Rutkowska-Hinc et al. found significant differences in subarachnoid fluid in the optic nerve sheath between DON and TAO without DON, suggesting that the optic nerve sheath may be an image marker for detecting DON in the future (25). Wu et al. assessed changes in the optic nerve and cerebrospinal fluid (CSF) in the optic nerve sheath in DON patients by using the iterative decomposition of water and fat with echo asymmetry and least-squares estimation-T2-weighted images (IDEAL-T2WI) sequence. They found that increased water fraction(WF) of the optic nerve and optic nerve subarachnoid space are promising and easily accessible radiological markers for diagnosing DON, providing help for early detection and timely treatment of DON by clinicians (27). In order to detect DON in patients with TAO, Liu et al. selected multi-parametric diffusion tensor imaging of the optic nerve for measurement. Using diffusion tensor imaging (DTI), Liu et al. found that compared with non-DON group, the DON group showed decreased fractional anisotropy and increased mean, axial, and radial diffusivity of the intra-orbital optic nerve. This suggests that DTI is a promising technique in assessing microstructural changes of optic nerve in patients with DON, and it facilitates differentiation of DON from non-DON eyes in patients with TAO (30). This team also applied functional MRI to explore the changes in brain function in patients with DON and found that the regional homogeneity (ReHo) of the left islet and the right superior temporal gyrus in patients with DON was lower than that in non-DON patients. The ReHo index can be used as a diagnostic biomarker for the discovery of DON (31).Hu et al. found that T2 relaxation time (T2RT) values calculated by T2 mapping can detect disturbance in the intraorbital optic nerve in patients with TAO, especially active TAO, before DON develops (32). T2 mapping has a potential for noninvasive evaluation of optic nerve changes in patients with TAO. Zhang et al. demonstrated that MRI 3D reconstruction based on volume rendering can be used to evaluate DON, and EOM enlargement could serve as a predictor of visual function in DON patients (33). Hu et al. showed that orbital MRI and intracranial visual pathway diffusion kurtosis imaging can both assist in diagnosing dysthyroid optic neuropathy. Combining orbital MRI and intracranial visual pathway diffusion kurtosis imaging optimized the diagnostic efficiency of dysthyroid optic neuropathy (34). These new imaging findings bring new insights for the precise diagnosis and treatment of DON, which is conducive to improving the quality of life and prognosis of patients.

### 2.2 Application of MRI in the activity evaluation

The natural course of TAO includes active stage and inactive stage. The active phase is characterized by inflammatory swelling of orbital fat and EOMs, leading to soft tissue congestion. During the inactive phase, inflammation subsides and is replaced by fibrosis of the extraocular muscles and the retroorbital tissues. Clinically, active phases of inflammation usually respond well to immunosuppressive therapy, while inactive phases of fibrosis usually require surgical intervention. Therefore, it is important to accurately distinguish the active and inactive stages of TAO and quantitatively detect orbital inflammation and fibrosis for the selection of appropriate treatment. CAS is a common index of activity staging based on typical inflammatory manifestations; however, this measure is less sensitive to subclinical patients. MRI is another way to evaluate the activity of TAO. Orbital MRI is accurate and can reflect the pathological changes of TAO such as inflammation, steatosis and fibrosis. Previous studies have confirmed that orbital MRI combined with CAS can improve the sensitivity of detection of disease activity. In recent years, a variety of new MRI sequence parameters have been used for the differentiation of TAO activity staging (**Table 2**).

**Table 2:** MRI sequences applied in the differentiation of Active Staging of TAO.

Active Staging	MRI Sequence	MRI parameter	Research findings
Active phase	T2WI/Dixon	SIR	A cut-off SIR value of > 2.9 in the inferior rectus could be applied to evaluate the active stage of TAO{Ge, 2021 #12}.
	T2 Mapping	T2RT	T2RTmean ≧ 74.295 could be a diagnostic cut-off for judging TAO activity{Li, 2023 #6}.
	DWI	ADC	The cutoff value of pretreatment n-ADC was 1.780 to detect stages{Liu, 2021 #14}.
Inactive phase	T1 Mapping	T1RT/ECV	ECV>48.1% can be used as the critical value for screening inactive TAO{Ma, 2022 #15}.
	MTI	MTR	Patients with active TAO showed significantly lower MTRs than those with inactive TAO{Hu, 2022 #16}.

T2WI, T2-weighted images; DWI, diffusion-weighted imaging; MTI, Magnetization transfer imaging; SIR, signal intensity ratio; T2RT, T2 relaxation time; ADC, apparent diffusion coefficient; T1RT, T1 relaxation time; ECV, extracellular-volume; MTR, magnetization transfer ratio; TAO, thyroid associated ophthalmopathy.

#### 2.2.1 Application in active phase

The active stage of TAO is characterized by vascular dilatation and inflammatory cell infiltration with orbital tissue edema. Orbital MRI can be used to identify inflammation and edema. Multiple MRI modalities have been utilized to assist in identifying TAO activity.

**Figure 1:**
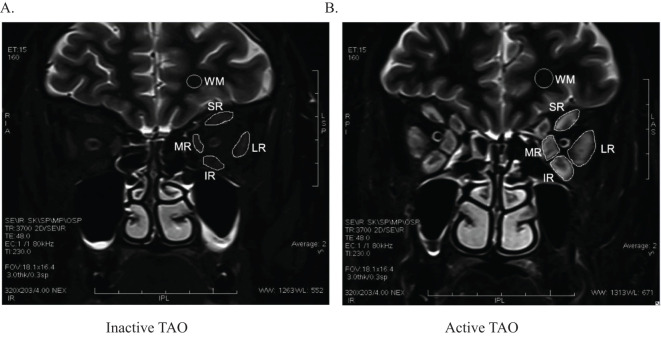
Measurement of signal intensities of extraocular muscles on STIR imaging in one inactive eye **(A)** and one active eye **(B)**. The signal intensity in each muscle was normalized to that in white matter of the same slice. SR, superior rectus; IR, inferior rectus; LR, lateral rectus; MR, medial rectus. WM, White matter{Ge, 2021 #9}.

Traditional T2-based signal intensity ratio (SIR) or relaxation time (T2RT) imaging is the most fundamental and widely recognized method, and is regarded as a powerful approach for differentiating disease activity (35, 36).The pulse sequence of T2 can estimate the water content of tissues. When examining the EOMs, normal T2 might imply fibrotic disease with low water content, while prolonged T2 might suggest ongoing inflammation with tissue oedema. Hoh et al. calculated the SIR of EOMs and temporalis muscles in TAO patients by STIR(short inversion time inversion recovery) sequence, which was higher than that in healthy controls and positively correlated with Werner activity scores (37). Another study on STIR MRI in patients with TAO also confirmed this finding and demonstrated a temporal correlation between SIR of the EOM with the most severe inflammation and CAS (35, 38). Several MRI studies have demonstrated that active TAO patients present with increased T2 signal intensity ratio (T2-SIR) of EOMs due to edema and indicated that it might be helpful in the diagnosis and staging of TAO (39–41). Higashiyama et al. confirmed that the SIR of orbital fat had a significant positive correlation with the CAS, and the measurements of SIR in orbital fat may be useful in evaluating the activity in tissues of TAO patients (42). Ge et al. found that the signal intensity of extraocular muscles on STIR sequence was a good predictor for TAO activity (**Figure 1**). A cut-off SIR value of > 2.9 in the inferior rectus could be applied to evaluate the active stage of TAO (43).A study based on 3-T magnetic resonance imaging to measure the lacrimal gland (LG) signal value and then compare it with the temporalis muscle to obtain SIR showed that the lacrimal gland SIR differs between active and inactive TAO groups, which is helpful for the diagnosis and staging of TAO (41). Dixon MRI is a fat-suppressed technique that assesses chemical shift analysis and can directly differentiate fat from water. Pu et al. found that dixon MRI-based parameters of EOMs, LGs, and intraorbital fat (IF) are useful for differentiating active from inactive TAO. Compared with inactive TAOs,active TAOs demonstrated significantly greater EOM-SIR,IF-SIR,LG-SIR. The integration of multiple parameters can further improve staging performance (44). Chen et al. discovered that two-point Dixon T2WI showed significantly higher overall image quality score, fat suppression (FS) uniformity score as well as EOM-SIR value than fat-sat T2WI. When integrating Dixon-EOM-SIR ≥3.32 and Dixon-IF-WF≥0.09, improved staging efficiency and specificity could be achieved (area under the curve, 0.872; specificity, 97.1%) (45). Therefore, using multiple orbital MRI sequences to measure the SIR of retroorbital tissues such as EOM and IF in patients with TAO is helpful for the differentiation of disease activity staging.

T2 mapping based on multi-echo spin-echo pulse sequences which quantifies native T2RT is a technique used for enabling visualization as well as quantification of water content and collagen structure, reflecting the histopathological changes of certain diseases (46). The T2RT reflects the water content of the tissue and is used as a way to assess the degree of inflammatory edema (47). T2 mapping sequence can not only quantitative measure T2RT to reflect the inflammation status, but also qualitatively evaluate the EOMs and adipose tissue. Das et al. found that T2RT of EOMs in TAO patients tended to increase in the active stage, which was positively correlated with CAS (48). Hou et al. measured T2RT of TAO by three dimensional (3D) T2-mapping MR sequence, and the results showed that CAS combined with 3D T2-mapping MR imaging could improve the sensitivity of detection of active TAO so as the prediction and evaluation of the response to methylprednisolone treatment (49). Li et al. confirmed that T2RTmean was independently associated with TAO active periods and had good diagnostic performance (area under ROC curve = 0.736, sensitivity 70.7%, specificity 69.3%). T2RTmean ≧ 74.295 could be a diagnostic cut-off for judging TAO activity (sensitivity 55.3%) (11).Jiang et al. found that there were significant differences in MRI quantitative parameters of LG between healthy controls and TAO patients, and the combination of the T2-mapping value of LG and clinical indicators improved the stage prediction of TAO compared to CAS (50). Another study demonstrated that the novel models combining LG T2RT and the absolute reduction in T1-relaxation time values showed excellent predictive performances in diagnosing TAO, while T2RT of LG showed practical utility for staging TAO (51).The above studies show that the T2RT value of T2 mapping is an independent factor for effectively predicting the activity of TAO. Combining the relevant parameters of other techniques is helpful to improve the diagnostic performance of patients with active TAO.

MRI with diffusion-weighted imaging (DWI) is another noninvasive technique in the evaluation of multiple diseases, due to its ability to detect the microscopic movements of water at the cellular level by using apparent diffusion coefficients (ADCs) to quantify the diffusion of water molecules in tissues. Kilicarslan et al. found that the increased ADC values of the EOMs in patients with TAO indicated that EOMs damage begins at a very early stage before being detected on routine orbital MRI, suggesting that DWI could detect EOMs involvement in the early stage of the disease (52). Recently, diffusion imaging with the simultaneous ADC and fractional anisotropy values has also shown considerable potency in differentiating active from inactive TAO, owing to the ability to quantify the magnitude and directionality of water diffusion (53, 54). Liu et al. found that T2-SIRs and normalized ADC (n-ADC) values of the involved EOMs are valuable MRI indicators of the inflammatory activity in TAO, and n-ADC values could be promising predictive factors in the detection of stages of diseases when combined with CAS. The cutoff value of pretreatment n-ADC was 1.780 to detect stages with specificity of 93.7% and sensitivity of 48.3% (55). Therefore, ADC can semi-quantitatively assess the inflammation and edema of the EOMs in patients with TAO, which has certain clinical significance for predicting the activity stage of TAO.

In conclusion, by using different orbital MRI sequences to measure SIR,T2RT, and ADC parameters, not only tissue swelling can be detected, but also the activity of inflammation can be objectively quantified, thereby predicting the response to immunosuppressive therapy.

#### 2.2.2 Application in inactive phase

The pathology of TAO in the inactive stage is characterized by fibrosis of the posterior orbital soft tissues such as the EOMs. MRI can quantitatively analyze the extracellular components of soft tissues and is considered a reliable noninvasive tool for evaluating histopathology. Several MRI modalities have been used to evaluate fibrosis.

The T1 mapping MRI, which enables to measure T1 relaxation time (T1RT), has achieved good performance to examine fibrotic changes in conditions such as cardiomyopathy, liver fibrosis, and chronic pancreatitis (56–58). This sequence has recently been used in orbital scan to assist clinical assessment of TAO, concluding that the T1RT values of EOMs were significantly lower in the inactive TAO patients compared with the active cases (59). A cross-sectional study using T1 mapping MRI to predict refractory diplopia in patients with inactive TAO concluded that this novel sequence may play a role in evaluating extraocular muscle fibrosis (60). Since native T1 is a complex parameter to interpret, a T1-derived index extracellular-volume (ECV) was generated for assessment of fibrosis. Ma et al. found that the ECV parameter on T1 mapping MRI was a reliable tool to quantify EOMs fibrosis. The performance of ECV (cutoff > 48.1%) to screen out extraocular muscle fibrosis in inactive TAO was 60.9% sensitivity and 93.3% specificity. It provides insights for the non-invasive assessment of orbital pathological changes in TAO (61). The T1RT value obtained based on T1 mapping can evaluate the pathological changes of the degree of fibrosis in patients with inactive TAO, suggesting its value in evaluating the pathological tissue changes at different stages of TAO, and providing a theoretical basis for further clarifying the staging of TAO and the selection of treatment methods in clinical practice.

Magnetization transfer (MT) imaging (MTI), an advanced MRI technique, can provide additional information about the pool of water bound to macromolecules, such as collagen, in the involved tissues (62). The MT effect can be expressed quantitatively using the magnetization transfer ratio (MTR), indirectly reflecting the macromolecular concentrations in an aqueous physiological environment (63). The technique has already been successfully applied to assess fibrosis pattern in cardiac tissue, degree of intestinal fibrosis in Crohn’s disease, and renal fibrosis in scarred kidneys (64–66). Hu et al. investigated the feasibility of using MT magnetic resonance imaging for evaluating patients with TAO, and compared with conventional fatsaturated T2-weighted and DWI. They found that patients with active TAO showed significantly lower MTRs and higher SIRs and ADCs than those with inactive TAO; suggesting that MT imaging could potentially be used as a noninvasive method for differentiating the activity of TAO and predicting CAS, thereby offering added value to conventional SIR and ADC (67).

Ollitrault et al. used the area with low signal in both the Dixon T2WI water map and in T2WI as a marker of EOMs fibrosis (68). Enlargement of EOMs with normal T2RT was used as the basis for determining chronic fibrosis (69). However, these methods do not quantitatively determine the extent of fibrosis, and diagnosis based on such imaging is highly subjective, so the clinical significance of this approach is unclear.

Therefore, for some patients with inactive TAO, multiple new sequences such as T1 mapping and MTI can be adopted to evaluate the degree of fibrosis of the EOMs and be used to assist in differentiating the active stage of the disease.

## 3 Application of MRI in the treatment of TAO

2021 EUGOGO Clinical Practice Guidelines recommend intravenous glucocorticoids(IVGC) for the treatment of moderate-to-severe active TAO (8). Clinically, the therapeutic effect is often evaluated according to the changes in patients’ CAS score or severity grading. Objective data obtained from orbital MRI can also be used to evaluate the therapeutic effect. Active TAO usually responds well to immunosuppressive therapy, while inactive TAO requires surgical intervention. MRI can be used to evaluate the disease activity, so it can predict the therapeutic effect according to the quantitative analysis results of MRI and help physicians select the most appropriate treatment schedule.

### 3.1 Application of MRI in the evaluation of treatment efficacy

High-dose systemic glucocorticoids have potent anti-inflammatory and immunosuppressive effects that have been applied successfully for the management of moderate-to-severe and active TAO (70). MRI can be used to evaluate the efficacy before and after glucocorticoids therapy. A study using ADC and CAS to evaluate the effects of 4-week and 12-week IVGC therapy showed higher changes in ADC in the 4-week group than in the 12-week group, suggesting greater improvement in inflammation in the 4-week group (71). Higashiyama et al. evaluated the efficacy of methylprednisolone pulse therapy in TAO by using MRI SIR (72). MRI measured the signal intensity of EOMs and white matter of brain, and calculated SIR of EOMs and white matter of brain. They found that SIR decreased significantly after treatment compared with that before, suggesting improvement of inflammation of EOMs. Another study was conducted by MRI to evaluate the changes of orbital tissue volumes and proptosis after methylprednisolone pulse therapy in TAO (15). The cross-sectional areas of the whole orbit, bony orbital cavity, six EOMs, eyeball, and optic nerve were measured by MRI. The volume of each tissue was calculated by multiplying the sum of the crosssectional areas by the slice increment. The volume of orbital fatty tissue was calculated by subtracting the volumes of the total EOMs, eyeball, and optic nerve from the whole orbital. And the results showed that the mean volume of total EOMs was significantly decreased after treatment, while the mean volume of orbital fatty tissue and proptosis value were not significantly decreased. The results may be related to the patient’s condition and the different mechanisms of the orbital fatty tissue and EOMs in patients wih TAO (73). And they speculated the reason why proptosis was not significantly decreased after treatment was that the volume of orbital fatty tissue was not significantly decreased. Tachibana et al. evaluated the activity of TAO and the response to IVGC using T2RT and CAS, concluded that CAS and maxT2RT showed significant positive correlation (10).Hu et al. confirmed that dynamic contrast-enhanced (DCE)-MRI derived model-free and model-based parameters of EOMs can assist in the evaluation of TAO. In particular, time to peak(TTP)-min and the volume transfer constant(Ktrans)-mean were independent predictors of the response to GCs (P = 0.023 and 0.004, respectively), uniting which could determine the response to GCs with decent performance (AUROC = 0.821) (74). Chen et al. explored the association between three-dimensional fast spin echo with 2-point Dixon-based fat suppression (3D-FSE-Dixon) based parameters and methylprednisolone pulse therapy(MPPT) efficacy in active moderate-to-severe thyroid-associated ophthalmopathy, found that higher water fraction of EOMs demonstrates better MPPT efficacy for TAO (75).

Teprotumuma and rituximab are also recommended for the treatment of moderate-to-severe and active TAO. Jain et al. analysed volumetric and inflammatory changes on orbital imaging prior to and after teprotumumab treatment in six TAO patients. They measured SIR of EOMs by MRI, and calculated 3D volume of EOMs, orbital fat, and bony orbit. The result showed that total EOM volume within each orbit was markedly reduced post-teprotumumab in all patients, total orbital fat volume was also reduced in 11 of 12 studied orbits, and EOM SIR was also reduced (76). Wang et al. studied the curative effect of rituximab to treat 7 patients with TAO using MRI, found the thickness of EOM with maximum signal intensity and EOM SIR were both significantly decreased after treatment (77).

Retrobulbar radiotherapy is a safe and effective treatment for progressive TAO. Yu et al. assessed the inflammation of EOMs in patients with TAO before and after radiation therapy by evaluating the ADC values in DWI images. They found that the ADCs were significantly decreased after treatment. There was a statistically significant correlation between the mean ADCs and the CAS in each patient with TAO both before and after treatment. This suggested the ADC values of EOMs can be exploited as a quantitative indicator to monitor the therapeutic responses of patients with TAO (78).

### 3.2 Application of MRI in predicting treatment efficacy

At present, MRI can not only be used for the diagnosis and activity evaluation of TAO, but also can assist clinicians in predicting the treatment efficacy through quantitative analysis of MRI parameters, as proved by several studies. Zhai et al. evaluated the performance of baseline clinical characteristics and pretherapeutic histogram parameters derived from T2 mapping of EOMs in the prediction of treatment response to IVGC therapy for active and moderate-to-severe TAO and investigated the effect of fat-suppression (FS) in T2 mapping in this prediction. Results showed that an integrated combination of disease duration, histogram parameter FS- 95th percentile and Fs-kurtosis was a potential predictor of treatment response to IVGC in patients with active and moderate-to-severe TAO, and FS T2 mapping was superior to conventional T2 mapping in terms of prediction (79). Hiromatsu et al. confirmed that the SI of EOM in patients with good response to methylprednisolone pulse therapy was significantly higher than that in patients without good response (80). Yokoyama et al. also confirmed that MRI-T2 SI of EOM in pretreatment showed a significant correlation with reduction in EOM volume for therapeutic effect, suggesting that T2-SI in pretreatment can be used as one of the reliable parameters to predict the therapeutic outcome of treatment (81). Another study found that T2WI-derived radiomics of EOMs combined with disease duration predicted the response of TAO patients to glucocorticoid therapy (82). Tachibana et al. used MRI maxT2RT and CAS to evaluate TAO activity and predict the response to IVGC therapy, and found that 20 patients whose activity was evaluated only by MRI had significant improvement after IVGC. They concluded that orbital MRI combined with CAS could improve the sensitivity of detection of active disease and the prediction of the response to IVGC (10).

## 4 Conclusion

In conclusion, MRI plays a very important role in the whole clinical diagnosis and treatment of TAO patients, which can not only assist in disease diagnosis, activity and efficacy evaluation, but also predict treatment response and help clinicians select the best treatment plan. With the development of MRI new technology and analysis software, various new sequences and quantitative analysis parameters are used for TAO. The appearance of DWI, T1 mapping, T2 mapping, MTI, etc. provides assistance in judging the pathological changes such as edema, adiposis and fibrosis of the retroorbital tissues of TAO. In order to further improve the clinical MRI assistance in TAO diagnosis and treatment, it is very important to establish a quantitative parameter combination model composed of necessary sequences, which should be realized by multidisciplinary physicians in the future.
